# Fetal Diagnosis of Hypoplastic Left Heart Syndrome With Restrictive Atrial Septum—Atrial Septal Morphology, Associated Lung Disease and Outcomes

**DOI:** 10.1002/pd.70000

**Published:** 2025-11-03

**Authors:** Sofie Dannesbo, Gloria Ho, Mike Seed, Vitor Guerra, Rajiv Chaturvedi, Andrew C. Cook, Niels Vejlstrup, Kasper Iversen, Henning Bundgaard, Lindsay R. Freud

**Affiliations:** ^1^ Labatt Family Heart Centre The Hospital for Sick Children University of Toronto Toronto Ontario Canada; ^2^ Department of Cardiology The Heart Centre Copenhagen University Hospital Rigshospitalet Copenhagen Denmark; ^3^ Faculty of Health and Medical Sciences University of Copenhagen Copenhagen Denmark; ^4^ Ted Rogers Computational Program Peter Munk Cardiac Centre University Health Network University of Toronto Toronto Ontario Canada; ^5^ Institute of Cardiovascular Sciences University College London London UK; ^6^ Department of Cardiology Copenhagen University Hospital Herlev Copenhagen Denmark

**Keywords:** fetal echocardiogram, fetal MRI, hypoplastic left heart syndrome, pulmonary lymphangiectasia, restrictive atrial septum

## Abstract

**Objective:**

Fetuses with hypoplastic left heart syndrome (HLHS) and restrictive/intact atrial septum (RAS) have high mortality, partly due to pulmonary lymphangiectasia (PL). This study aimed to characterize atrial septal morphology in fetuses with HLHS and RAS and evaluate the impact of fetal intervention on PL and outcomes.

**Method:**

We retrospectively reviewed clinical data, fetal echocardiograms, and MRIs from all fetuses with HLHS/HLHS‐variants and RAS over 20 years. RAS was defined as pulmonary venous forward‐to‐reverse VTI ratio ≤ 5:1.

**Results:**

We identified 54 fetuses (gestational age of 24.2 weeks; 21.2–28.1) with HLHS (81%) or HLHS variant (19%) and RAS. Most had a hypoplastic left atrium (LA, 80%). Four atrial septal morphology patterns were identified. PL was present among all patterns and associated with lower VTI ratio (*p* = 0.046). Mean VTI ratio improved in fetuses who underwent atrial septal intervention (*n* = 12), compared to those without (*p* = 0.001). Among five fetuses with pre‐intervention PL and serial MRIs, three improved. Seventeen neonates were live‐born with intention‐to‐treat; 6‐months mortality was 38% with no difference by morphology pattern, PL, or fetal intervention.

**Conclusion:**

Most fetuses with HLHS and RAS had hypoplastic LA. VTI ratio correlated with PL on fetal lung MRI, and most improved with fetal atrial septal intervention.

## Summary

- What's already known about the topic?
  ◦ Hypoplastic left heart syndrome with restrictive or intact atrial septum has high mortality, partly due to in utero development of pulmonary lymphangiectasia.
  ◦ Fetal atrial septal invention may modify the high mortality risk; however, patient selection is challenging owing to highly heterogeneous but poorly characterized atrial septal morphology.
- What does this study add?
  ◦ We identified four different atrial septal morphology patterns in fetuses with hypoplastic left heart syndrome and variants with restrictive or intact atrial septum. Contrary to a prior study, we found that most fetuses had a hypoplastic left atrium.
  ◦ Pulmonary venous VTI ratio correlated with pulmonary lymphangiectasia on fetal lung MRI, and most improved with fetal atrial septal intervention.

## 1 Introduction

Restrictive or intact atrial septum (RAS) is a high‐risk anatomical feature in fetuses with hypoplastic left heart syndrome (HLHS) [1]. In HLHS and other single ventricle variants with lack of left ventricular inflow, the circulation requires an adequate atrial communication for pulmonary venous return. When the atrial communication is inadequate, pulmonary vascular congestion may evolve in utero and lead to abnormal development of the pulmonary vasculature and parenchyma, including pulmonary lymphangiectasia (PL) [2, 3]. Postnatally, RAS precludes oxygenated blood from reaching the systemic circulation, leading to severe neonatal instability [4]. As a result, mortality in this patient population is reported to be the highest among single ventricle patients [5, 6, 7].

Fetal echocardiography is an essential tool for diagnosing and monitoring atrial restriction in this high‐risk population. Pulmonary venous Doppler patterns are particularly helpful in assessing the degree of restriction [4, 8, 9, 10, 11]. In addition, fetal magnetic resonance imaging (MRI) may be used to assess lung disease, particularly PL, in patients with HLHS and RAS in utero [12, 13, 14, 15, 16, 17]. These diagnostic modalities may facilitate the selection of candidates for fetal atrial septoplasty or atrial septal stenting [17, 18, 19, 20, 21, 22] or help to risk‐stratify patients for delivery planning and postnatal management.

However, atrial septal morphology is heterogeneous in this patient group and has not been well‐characterized in fetal life. In 1999, Rychik et al. [23] described 3 categories of atrial septal morphology in 18 infants with HLHS and intact atrial septum. Most of these infants had large left atria (LA), which is not reflective of our experience in the fetal period. Others have described leftward‐posterior deviation of septum primum in up to 64% of neonates with HLHS [24, 25, 26], as well as more complex morphology [27]. A complete evaluation of atrial septal morphology in fetuses with HLHS and RAS has not been undertaken and may help refine decision‐making for pre‐ or postnatal atrial septal intervention.

Our study aimed to comprehensively describe the atrial septal morphology in a large series of fetuses with HLHS and RAS. We also sought to assess associations between fetal echocardiographic and MRI findings, to examine the effect of fetal intervention, and to explore factors related to perinatal outcome in this high‐risk population.

## 2 Methods

### 2.1 Study Design and Cohort

In this retrospective cohort study, we included all fetuses with HLHS and HLHS variants with RAS seen at the Hospital for Sick Children (HSC) in Toronto, Canada from April 2004 to June 2024 with at least one fetal echocardiogram available for review. HLHS variants included single ventricle lesions with lack of left ventricular inflow, such as double outlet right ventricle with mitral atresia. Patients were initially identified from the Fetal Cardiac Program database. Subsequently, the fetal echocardiograms were reviewed. We only included patients who had an averaged pulmonary venous flow forward‐to‐reverse velocity time integral (VTI) ratio ≤ 5:1.

### 2.2 Ethics

The study complies with the Declaration of Helsinki and was approved by The HSC Research Ethics Board (REB no.: 1000081322) with a waiver of informed consent.

### 2.3 Clinical Data Collection and Definitions

Study data were collected and managed using Research Electronic Data Capture (REDCap) hosted by HSC [28, 29]. Clinical data were collected by review of medical records. Prenatal demographic data included maternal age, estimated due date, family history of congenital heart disease, consanguinity, and the presence of extracardiac malformations and/or genetic diagnoses. Fetal MRIs were evaluated for signs of pulmonary disease, including nutmeg pattern consistent with PL or the presence of pleural effusions. Pregnancy outcome was categorized as elective termination of pregnancy, fetal demise, or live birth. For live‐born patients, we collected gestational age at birth, sex, and birth weight. The family's decision to pursue active management or compassionate care was noted. For patients who had active management (intention to treat), age and type of neonatal interventions were noted, as well as date of last follow‐up or death. Fetal atrial septal intervention data were collected, if performed. Fetal atrial septal intervention at our institution is undertaken for fetuses with HLHS and severely restrictive or intact atrial septum, based on both morphologic and physiologic features as previously described [17]. The presence of PL is not considered a contradiction. Each case is reviewed by a multidisciplinary team of experts and with the expectant patient and family prior to proceeding.

### 2.4 Fetal Echocardiograms

Fetal echocardiograms were independently reviewed. The first fetal echocardiogram was analyzed for all patients. If multiple fetal echocardiograms were available, the following were also analyzed: studies performed the same day, or in closest proximity to, fetal MRI studies performed; the last study performed prior to fetal intervention (if performed); and the last study prior to pregnancy outcome.

We classified the subtype of standard forms of HLHS as mitral stenosis/aortic stenosis, mitral stenosis/aortic atresia, or mitral atresia/aortic atresia. HLHS variants included fetuses with double outlet right ventricle with mitral atresia and left heart hypoplasia with significant ventricular septal defects. The following were noted: mitral regurgitation, tricuspid regurgitation, right ventricular function, and fluid collections or hydrops. The presence and location of an accessory channel from the LA was also documented. In our series, we do not refer to it as a decompressing channel since, based on our inclusion criteria, it did not adequately decompress the LA. We also elected not to call it a levoatrial cardinal vein due to anatomic variability.

The following pulmonary venous flow measurements were performed: forward flow VTI, reverse flow VTI, and reverse flow duration (msec). All flow measurements were performed three times and on both a right and a left pulmonary vein where feasible. Forward‐to‐reverse VTI ratios were calculated for both sides, and the average of the three measurements was documented. A VTI ratio ≤ 5:1 but > 3:1 was considered moderately restrictive and a VTI ratio ≤ 3:1 was considered severely restrictive [8].

Atrial morphologic features were assessed both quantitatively and qualitatively. Quantitative measurements included the combined atrial diameter of the right atria (RA) and LA and the LA diameter alone, as measured from a four‐chamber view. Qualitative characteristics included the size of the LA relative to the RA, defined as hypoplastic, normal or dilated; the thickness of the atrial septum (thin or moderately to severely thickened); presence of rightward bulging of the atrial septum; leftward‐posterior deviation of septum primum; whether there was visible color Doppler flow across the atrial septum, and if so, whether multiple interatrial communications were present. A description of the morphologic characteristics was made for each patient. Based on these descriptions and the independent review of the fetal echocardiograms by two reviewers (SD and LF), four different morphology patterns were identified. If categorization was unclear, the fetal echocardiographic images were re‐reviewed by the two experts to arrive at a consensus.

### 2.5 Neonatal Echocardiograms

When available, the first postnatal echocardiogram prior to neonatal intervention was analyzed for the same atrial morphologic features. The pre‐ and postnatal findings were compared.

### 2.6 Statistical Analysis

Clinical characteristics were summarized using descriptive statistics. Categorical variables are presented as frequencies and percentages, and continuous variables are presented as median and interquartile range (IQR). Between‐group differences in categorical and continuous variables were assessed using Fisher's exact tests and Wilcoxon rank‐sum tests, respectively. Differences between forward‐to‐reverse VTI ratio at first and last fetal echocardiogram by fetal atrial septal intervention were estimated using generalized estimated equations, with standard errors and 95% confidence intervals estimated using the robust sandwich estimator. Wilcoxon rank‐sum test was applied to assess the between‐group difference in the change in VTI ratio from first to last fetal echocardiogram. The Kaplan–Meier survival method was used to estimate 30‐days and 6‐months transplant‐free survival rates, with between‐group differences assessed using log‐rank tests. Significance level was set at *p* < 0.05.

## 3 Results

A total of 54 fetal patients met our inclusion criteria, presenting at a median gestational age of 24.2 (21.2–28.1) weeks for their first fetal echocardiogram. Table 1 displays the characteristics of the cohort. Genetic testing was performed in 28 (52%) fetuses of which four (14%) had abnormal findings: 22q11.2 deletion syndrome (*n* = 2), Noonan syndrome (*n* = 1), and mosaic Turner syndrome (*n* = 1). Of the seven (13%) with a family history of congenital heart disease, two (29%) had known first‐ or second‐degree relatives with a bicuspid aortic valve.

**TABLE 1:** Prenatal demographic characteristics.

Prenatal characteristics	*N*	Median (IQR) or *n* (%)
Maternal age, years	54	30.4 (26.4–36.0)
Gestational age at first fetal echocardiogram, weeks	54	24.2 (21.2–28.1)
Extracardiac abnormalities	54	7 (13%)
Genetic diagnosis	28	4 (14%)
Consanguineous parents	54	4 (7%)
Family history of CHD	54	7 (13%)

Abbreviations: CHD, congenital heart disease; IQR, interquartile range.

### 3.1 Fetal Echocardiographic Findings

Table 2 summarizes the fetal echocardiographic findings. Most patients (*n* = 44, 81%) had standard HLHS. Moderate to severe mitral regurgitation was seen in four patients (7%), and a pericardial effusion was observed in six (11%). Approximately 1/5 of fetuses (*n* = 10, 19%) had an accessory channel draining from the LA, often superiorly toward the innominate vein or superior vena cava. Despite this finding (and per our inclusion criteria), the median pulmonary venous forward‐to‐reverse VTI ratio on the first study was 2.65:1 (1.62:1–3.64:1).

**TABLE 2:** Fetal echocardiographic findings in fetuses with HLHS/HLHS variant and RAS from the first study of gestation (*n* = 54) and last study prior to pregnancy outcome or fetal atrial septal intervention for patients with multiple studies (*n* = 23).

Variable	*N*	Median (IQR) or *n* (%)
Gestational age at first study, weeks	54	24.2 (21.2–28.1)
Gestational age at last study, weeks	23	32.3 (26.0–35.5)
HLHS phenotype	54	
Standard HLHS		44 (81%)
Mitral stenosis/aortic stenosis		6 (11%)
Mitral stenosis/aortic atresia		19 (35%)
Mitral atresia/aortic atresia		19 (35%)
HLHS variant		10 (19%)
Double outlet right ventricle/mitral atresia		4 (7%)
Other		6 (11%)
Pulmonary venous flow velocities		
Forward‐to‐reverse VTI ratio on first study in gestation	54	2.65 (1.62–3.64)
A‐wave duration on first study in gestation, msec	53	86.0 (79.0–92.7)
Forward‐to‐reverse VTI ratio on last study in gestation	23	2.45 (1.69–3.46)
A‐wave duration on last study in gestation, msec	21	82.7 (80.5–95.0)
Qualitative assessment of atrial dimensions	54	
Hypoplastic LA		43 (80%)
Near normal LA size		9 (17%)
Dilated LA		2 (4%)
Quantitative assessment of atrial dimensions		
LA diameter on first study in gestation, mm	53	6.2 (4.8–7.7)
Combined atrial diameter on first study of gestation, mm	54	18.4 (14.4–25.9)
LA‐to‐combined diameter ratio on first study in gestation	53	0.32 (0.28–0.38)
LA diameter on last study in gestation, mm	20	8.5 (74–11.1)
Combined atrial diameter on last study of gestation, mm	21	28.8 (21.7–32.5)
LA‐to‐combined diameter ratio on last study in gestation	20	0.34 (0.28–0.42)
Thickness of atrial septum	54	
Thin		8 (15%)
Moderate‐severely thickened		46 (85%)
Plane of atrial septum	54	
Normal plane of atrial septum		22 (41%)
Leftward‐posterior deviated atrial septum		32 (59%)
Rightward bulging of atrial septum		48 (89%)
Patency of atrial septum	54	
Intact atrial septum		12 (22%)
Patent interatrial communication(s)		42 (78%)
Multiple communications		7 (17%)
Other echocardiographic findings		
Mitral regurgitation ≥ moderate	54	4 (7%)
Pericardial effusion	54	6 (11%)
Accessory channel from LA	54	10 (19%)
Right ventricular function reduced ≥ moderate	54	0 (0%)
Tricuspid regurgitation ≥ moderate	54	2 (4%)

Abbreviations: HLHS, hypoplastic left heart syndrome; IQR, interquartile range; LA, left atrium; VTI, velocity time integral.

By qualitative assessment, most fetuses had a hypoplastic LA (*n* = 43, 80%). Only two patients had a dilated LA; both had severe mitral regurgitation. The atrial septum was moderate to severely thickened in the majority (*n* = 46, 85%). An intact atrial septum was observed in 12 (22%). Of the ones with an atrial communication (*n* = 42), seven had multiple communications.

### 3.2 Atrial Septal Morphology Patterns

**FIGURE 1:**
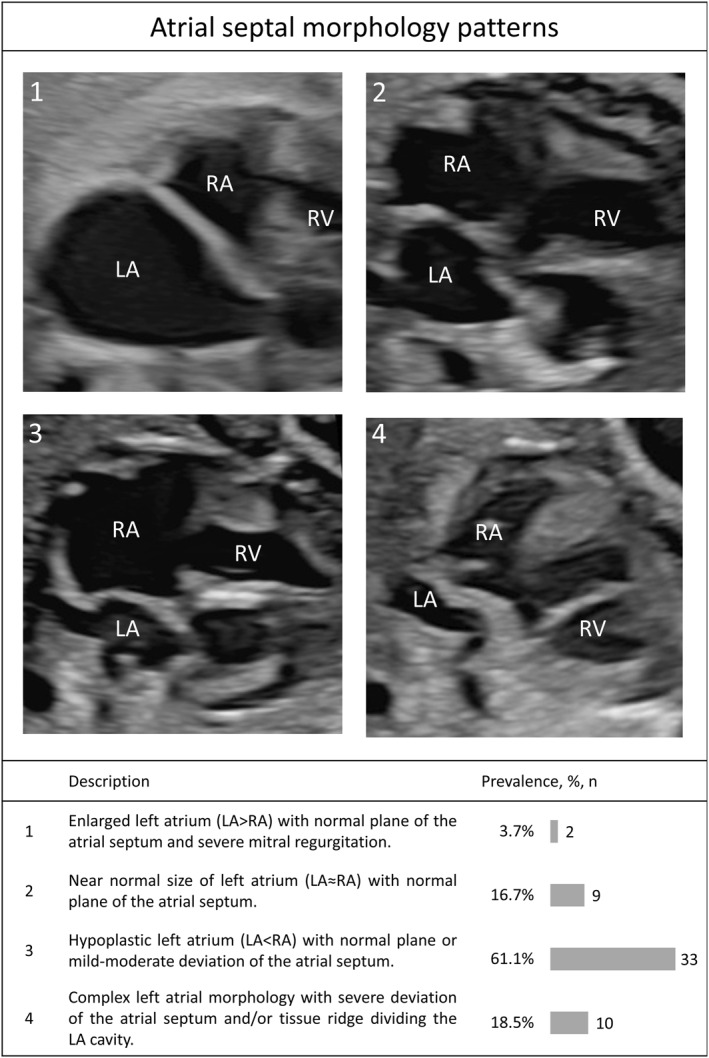
Atrial morphology patterns categorized into four types based on imaging characteristics. The four types (1–4) are displayed with (a) image examples from the fetal cardiac four‐chamber view, (b) short descriptions, and (c) bar‐plot showing distributions among the types. LA, left atrium; RA, right atrium; RV, right ventricle.

**FIGURE 2:**
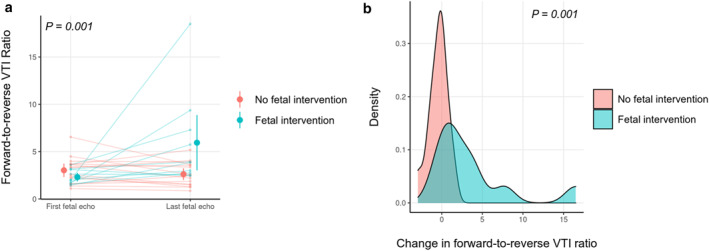
Forward‐to‐reverse VTI ratio over gestation. (a) Changes in forward‐to‐reverse VTI ratio between first and last echocardiogram in gestation in 23 fetuses with repeated fetal echocardiograms stratified by fetal atrial septal intervention; (b) Density plot showing significant change for the fetal atrial septal intervention patients. VTI, velocity time integral.

Based on the fetal echocardiographic findings, we identified four different atrial septal morphology patterns (Figure 1). Type 1 (Figure 1‐1) with a dilated LA was the least common type (*n* = 2, 3.7%) and existed only in the setting of severe mitral regurgitation. Type 2 (Figure 1‐2) appeared closest to normal atrial morphology, with a normal plane of the atrial septum and a near normal LA size (*n* = 9, 16.7%). The most frequent pattern was hypoplastic LA with a normal plane or mild‐moderate deviation of the atrial septum (Type 3 (Figure 1‐3), *n* = 33, 61.1%). Finally, Type 4 (Figure 1‐4) (*n* = 10, 18.5%) consisted of various complex morphologies, including a severely deviated atrial septum primum and/or tissue ridge dividing the LA cavity, potentially contributing to additional pulmonary venous obstruction. Further details are available in Supporting Information S1: Table S1.

An intact atrial septum was seen in all patterns, except Type 2. The 10 fetuses with accessory channels all had Type 3 or 4 morphology patterns. Accessory channels were seen most frequently in fetuses with complex patterns (Type 4) (40%). The numbers were too small to investigate associations with mortality. Further details are available in Supporting Information S1: Table S2.

### 3.3 Fetal Atrial Septal Intervention by Atrial Septal Morphology

In our cohort, 15 fetal atrial septal interventions were performed in 12 fetuses (22% of the cohort). In nine (60%) procedures, a stent was inserted. In two (17%) cases, intraprocedural fetal demise occurred. In another two (17%) cases, fetal demise occurred within 24 h of the procedure. Fetal atrial septal interventions were performed in fetuses with all four morphology pattern types. The two fetuses that received multiple interventions had Types 2 and 4, while the intraprocedural fetal demises occurred in fetuses with Types 1 and 3 (Supporting Information S1: Table S2).

### 3.4 Pulmonary Venous Flow Patterns

Based on the last echocardiogram in gestation or the last before fetal intervention, 33% (*n* = 18) of the cohort had findings suggestive of moderate atrial restriction and 67% (*n* = 36) of severe atrial restriction. Forward‐to‐reverse pulmonary venous VTI ratios by atrial septal morphology are presented in Supporting Information S1: Table S2.

Among fetuses who did not undergo atrial septal intervention (*n* = 42), the mean pulmonary venous forward‐to‐reverse VTI ratio was 3.0:1 (95% CI = [2.3:1–3.7:1]) on the first study and 2.6:1 (95% CI = [2.0:1–3.2:1]) on the last study in gestation. In contrast, the VTI ratio increased from a mean of 2.3:1 (95% CI = [1.8:1–2.7:1]) to 5.9:1 (95% CI = [3.0:1–8.9:1]) in fetuses who received atrial septal intervention (*n* = 12). The change in VTI ratio over gestation was statistically significant between fetuses who received atrial septal intervention and the ones who did not, *p* = 0.001 (Figure 2a,b).

In approximately half of the fetuses (*n* = 29), pulmonary venous forward‐to‐reverse VTI ratios were able to be measured on both the right and left sides. At the first study in gestation, 34% (10 of 29) fetuses had discordant measurements between the right and left sides. Specifically, six had findings suggestive of severe restriction on one side and moderate restriction on the other, while two had findings suggestive of moderate restriction on one side and non‐restrictive measurements on the other. The findings were more common at the time of the last study in gestation, whereby 44% (4 of 9) fetuses with bilateral measurements had discordance. The discordances were all between moderate and severe restriction, except for one patient in late gestation with a discordance between moderate and no restriction. No fetus at any time point had severe restriction on one side and no restriction on the other side (Supporting Information S1: Table S3).

### 3.5 Fetal Lung MRI Findings

A total of 23 (43%) fetuses had a fetal MRI performed at a median gestational age of 30.1 (24.9–32.9) weeks. Nine (39%) fetuses had abnormal findings: all had PL and one also had a pleural effusion. The median gestational age at MRI among the fetuses with PL (*n* = 9) was 32.7 (32.0–35.0) weeks as compared to 25.6 (24.4–30.3) weeks among fetuses with normal studies (*n* = 14), (*p* = 0.035). PL occurred in all atrial septal morphology types (Supporting Information S1: Table S2).

The fetal echocardiogram used for association with MRI findings was performed on the same day in 15 (65%) fetuses. In the remaining, the fetal echocardiogram was obtained within a median of 2 days (1–4) of the MRI. Fetuses with PL (*n* = 9) had significantly lower forward‐to‐reverse pulmonary venous flow VTI ratio compared to fetuses with no PL (*n* = 14) (1.51:1 (1.25:1–2.01:1) versus 2.81:1 (1.77:1–3.61:1), *p* = 0.046).

Seven fetuses had a repeat MRI later in gestation; five of whom had fetal atrial septal intervention between the two MRI studies. Of the five with post‐intervention MRIs, three had improvement, but not resolution, of PL; one developed PL on the post‐intervention study; and one remained with normal findings. Of the two other fetuses who had repeat MRIs, one had normal findings on both studies, and one had a normal study at 30 weeks and developed PL at 36 weeks. After the diagnosis of PL at 36 weeks, this fetus received a fetal atrial septal intervention in week 37.

Of the 23 fetuses who had MRIs, 13 were live‐born with intention of treat. The 6‐months mortality of fetuses with PL was three out of eight (38%) as compared to one out of five (20%) in fetuses without PL; however, this finding was not statistically significant due to the small sample size.

### 3.6 Pregnancy Outcomes and Surgical Management

Termination of pregnancy was elected in 21 (40%) of pregnancies. One pregnant patient left the country after diagnosis, and the pregnancy outcome was unknown. Of the 32 remaining pregnancies, fetal demise occurred in 7 (22%) pregnancies. Four fetal demises occurred in pregnancies where fetal intervention was performed (33%) compared to three in the pregnancies with no intervention (15%) (*p* = 0.38). Twenty‐five (78%) pregnancies resulted in live‐birth. Eight families elected for compassionate care after birth (32%), none of whom had a fetal intervention. The median gestational age at birth of those who had fetal intervention was 37.6 (35.8–38.8) weeks as compared to 38.4 (38.1–40.0) weeks in those without fetal intervention (*p* = 0.20). Additional neonatal characteristics are displayed in Table 3. Upon review of the neonatal echocardiograms, there were no changes in the categorization of the atrial septal morphology type.

**TABLE 3:** Pregnancy outcome, neonatal characteristics, and postnatal treatment of all fetuses with HLHS/HLHS variant and RAS.

	*N*	Overall	No fetal atrial septal intervention	Received fetal atrial septal intervention	*p*‐value
Pregnancy outcome		*n =* 54	*n =* 42	*n =* 12	
Unknown	54	1 (2%)	1 (2%)	0 (0%)	—
Termination	54	21 (40%)	21 (50%)	0 (0%)	—
Outcome for managed pregnancies	32				0.38
Fetal demise		7 (22%)	3 (15%)	4 (33%)	
Livebirth		25 (78%)	17 (85%)	8 (67%)	
Neonatal characteristics for live born		*n =* 25	*n =* 17	*n =* 8	
Compassionate care	25	8 (32%)	8 (47%)	0 (0%)	0.03
Sex, male	22	14 (64%)	9 (64%)	5 (62%)	1.00
Birth weight, kg	18	3.04 (2.84–3.34)	3.15 (2.84–3.42)	3.00 (2.96–3.31)	0.72
Gestational age at birth, weeks	21	38.4 (38.0–39.1)	38.4 (38.1–40.0)	37.6 (35.8–38.8)	0.20
Surgical course for intention to treat patients		*n* = 17	*n* = 9	*n* = 8	
Age at first intervention, days	16	2 (0–4)	2 (1–4)	1 (0–3)	0.55
Atrial septal intervention prior to stage 1	17	4 (24%)	1 (11%)	3 (38%)	0.29
Age, days	4	1 (1–1.25)	2	1 (1–1)	—
Stage 1/Norwood	17	17 (100%)	9 (100%)	8 (100%)	—
Age, days	17	4 (1–6)	4 (1–6)	4 (2–8)	0.88
Mortality for intention to treat patients		*n =* 17	*n =* 9	*n =* 8	
30‐day mortality	17	5.9%	0.0%	12.5%	0.29
6‐month mortality	17	37.3%	37.5%	37.5%	0.89

*Note:* Data are presented as *n* (%) or median (interquartile range).

Of the 17 neonates with intention to treat, 4 received a postnatal atrial septal intervention prior to Stage 1 (Norwood) surgical palliation (24%); 3 of these patients also had fetal atrial septal interventions. All 17 neonates underwent Stage 1 surgery. The median age at first neonatal intervention was 2 (0–4) days, and median age at Stage 1 surgery was 4 (1–6) days. There were no statistically significant differences in timing of first neonatal intervention or Stage 1 surgery between infants who did and did not receive fetal intervention (Table 3).

### 3.7 Mortality

The overall 30‐days and 6‐months mortality in the live‐born intention to treat cohort (*n* = 17) was 5.9% and 37.3%, respectively (Table 3). In this small cohort, there were no statistically significant differences in mortality by pulmonary venous VTI ratio ≤ 3:1 or > 3:1, presence of PL on fetal lung MRI, or fetal intervention.

## 4 Discussion

In this large cohort of fetuses with HLHS and HLHS variant with RAS, we present a complete categorization of atrial septal morphology and LA types, distinct from previous descriptions in newborns with HLHS and RAS [23]. We found that most fetuses had hypoplastic, rather than dilated, LA. Pulmonary venous Doppler tracings suggestive of significant restriction correlated with PL on fetal lung MRI. Moreover, fetal atrial septal intervention improved PL in a subset of patients. In the smaller subset of live‐born neonates with intention to treat, atrial septal morphology, pulmonary vein and fetal lung MRI findings, and fetal intervention did not correlate with mortality.

### 4.1 Atrial Septal Morphology

Based on our review of fetal echocardiograms, we identified four different patterns of atrial septal morphology. The first three types are based predominantly on LA size, which may stem from underlying patterns of embryonic development and/or abnormal loading conditions. The molecular pathways that led to HLHS, for example, may also be responsible for LA hypoplasia in a subset of patients. Abnormal flow from the ductus venosus in the setting of HLHS, aberrant left to right shunting at the foramen ovale, and/or mitral regurgitation may further contribute to pathology.

Consistent with our clinical suspicion, most fetuses with HLHS and HLHS variant with RAS had small LA: 61% were Type 3. An additional 18% were also hypoplastic in the setting of more complex septal deviation and additional tissue planes and/or muscular ridges in the LA (Type 4). While we had nearly 80% with hypoplastic LA, Rychik et al. [23] described hypoplastic LA in only 22%. In both of our cohorts, a dilated LA in the setting of mitral regurgitation was uncommon: 4% in our cohort and 11% in the historical neonatal cohort.

There are several possible explanations for the differences in our findings. Our study included 54 fetuses with HLHS or HLHS variant and defined atrial septal restriction by pulmonary vein Doppler patterns, the standard in fetal life. In contrast, the historical cohort was comprised of 18 live‐born infants, all with standard HLHS and an intact septum. While survival bias may have played a role in the historical live‐born cohort, we did not discern important mortality differences by atrial septal type. That being said, bias may have arisen due to the relatively small sample size. The findings in our cohort remained consistent from the second trimester through the neonatal period, which argues against evolution of LA size in the latter half of gestation. In fact, given that 80% of our cohort had a hypoplastic LA, a strong case could be made that the embryonic maldevelopment of the LA itself contributes to atrial septal restriction.

This notion may be further supported by the findings seen in the Type 4 fetuses, whereby complex morphologic features and LA hypoplasia were noted to occur together. Qasim et al. [27] recently described similar findings in fetuses with HLHS, which they named “labyrinthine‐cor” in a reference to the malformation being a type of cor‐triatriatum sinister. The authors found an increased mortality in those with “labyrinthine‐cor.”

Fetal atrial septal interventions were performed in fetuses of all atrial septal morphology types; however, one patient with Type 4 required multiple interventions to establish patency of an atrial communication. The technical challenges of fetuses with Type 4 atrial septae should be acknowledged and weighed carefully prior to offering fetal intervention. Severe hypoplasia of the LA, as seen in the Type 3 fetuses, may be another limitation to technical performance of the procedure. These are also important considerations for postnatal intervention. Mild to moderate LA hypoplasia does not imply that there is not significant LA hypertension worthy of decompression. Most of the fetuses in our cohort fell into this category based on their pulmonary vein Doppler findings, thereby challenging the postnatal dogma that “tense” LA are dilated.

### 4.2 Pulmonary Lymphangiectasia

PL is known to occur in fetuses with HLHS and RAS and has been shown to be a negative prognosticator [15, 16]. Similarly, in our series, 6‐months mortality was nearly twofold in those with PL on the initial fetal lung MRI. This finding did not meet statistical significance, likely due to a combination of the small sample size and our center's strategy of offering fetal intervention for RAS, even in the presence of PL. One‐third of the patients in our series who had successful intervention did, in fact, have improvement in the appearance of PL on serial lung fetal MRIs. This finding serves to substantiate the rationale for fetal intervention in this population at high‐risk of mortality.

There was a significant association between low pulmonary venous VTI ratio and PL on MRI. Since fetal MRI is not a widely available resource, this finding underscores that fetuses with low VTI ratios should be referred to tertiary fetal care centers for further evaluation. If possible, referral to a fetal cardiac intervention center would be ideal to further evaluate the maternal‐fetal dyad for candidacy and feasibility of LA decompression. Our study further highlights that, occasionally, there is discordance between right and left pulmonary vein measurements, and both sides should be routinely evaluated.

### 4.3 Mortality

While neonatal mortality in our fetal cohort was low (5.9%), 6‐months mortality remained substantial (37.3%). This figure is, however, lower than a contemporary series from a large non‐fetal intervention center in the UK. Ramcharan et al. [6] reported that 9 of 16 (56%) patients with HLHS and RAS patients did not survive the Norwood/Stage 1 in the neonatal period. In 2017, the International Fetal Cardiac Intervention Registry (IFCIR) reported a 61% neonatal mortality in fetuses with HLHS and RAS, with a modest, non‐significant survival advantage for those who underwent fetal intervention (*n* = 47, 44% vs. 33%). A recent meta‐analysis by Mustafa et al. [30] of 746 fetuses with HLHS and RAS, of which 123 underwent fetal atrial septal intervention, reported comparable neonatal mortality between intervention and non‐intervention patients. By 1 year of age, however survival in the IFCIR cohort was significantly better in the intervention group (59% vs. 19%) [31]. This finding underscores that longer term follow‐up of patients, ideally across institutions, remains essential to understand the natural and intervened upon history of fetal HLHS and HLHS variants with RAS.

### 4.4 Limitations

Our study was limited by its retrospective nature over a 20‐year period, during which time clinical practice evolved. While pulmonary vein Dopplers were widely available, fetal lung MRI data were limited to the more recent cohort. In addition, based on the quality of the fetal echocardiograms over the years and the 3D nature of the atrial septum, it was difficult to accurately quantify certain findings such as atrial septal thickness. Therefore, thickness was assessed qualitatively. More reliable and reproducible findings, such as LA size and complexity of atrial septal morphology, were used as the basis for our classification. All images were categorized by two experienced reviewers; however, interobserver variability was not formally ascertained. PL on fetal lung MRI was noted to be present or absent, since a grading scale does not currently exist. The live‐born intention to treat cohort comprised less than one‐third of the fetal cohort, precluding significant conclusions regarding outcomes. Nevertheless, the fetal cohort represents one of the largest and most well‐characterized, both by echocardiography and MRI, to date.

### 4.5 Conclusion

Among fetuses with HLHS and HLHS variants with RAS, the majority had hypoplastic LA and almost one‐fifth had complex atrial septal morphology. These findings remained consistent from the second trimester through neonatal life. There was a correlation between forward‐to‐reverse pulmonary venous flow VTI ratio on fetal echocardiography and the presence of PL on fetal lung MRI, and most patients improved with fetal atrial septal intervention. Although 6‐months mortality was substantial in our cohort, it was lower than other recently reported series, which may be due to our center's approach. Regardless of timing for atrial septal intervention, a nuanced understanding of atrial septal morphology and LA size may help guide clinical management and decision‐making in this high‐risk population.

## Conflicts of Interest

The authors declare no conflicts of interest.

## Ethics Statement

The study complies with the Declaration of Helsinki and was approved by The HSC Research EthicsBoard (REB no.: 1000081322).

## Consent

This retrospective study is based on data collected as part of standard clinical practice and approvedwith a waiver of informed consent

## Supporting information


Supporting Information S1

## Data Availability

The data that support the findings of this study are available from the corresponding author upon reasonable request.
