# Supplementary material for: Fetal Diagnosis of Hypoplastic Left Heart Syndrome With Restrictive Atrial Septum—Atrial Septal Morphology, Associated Lung Disease and Outcomes
Source: Prenat Diagn. 2025 Nov 3;46(2):183–92. doi: 10.1002/pd.70000 (PMC12880957; doi:10.1002/pd.70000)
Supplement: Supplementary file 1 — Supporting Information S1 [file PD-46-183-s001.docx]

**Supplemental Table S1**: Detailed description of atrial septal morphology in 10 fetuses with the complex morphology Type 4.

| **Patient** | **Description** |
| --- | --- |
| Patient 1 | Hypoplastic LA. Very deviated septum with inferiorly located patent atrial communication and additional tissue folds in LA. |
| Patient 2 | Hypoplastic LA. Deviated septum with superior patent atrial communication and several infoldings of the atrial wall in LA. |
| Patent 3 | Hypoplastic LA. Aneurysmal deviated septum with multiple fenestrations, and additional tissue folds in LA. |
| Patient 4 | Hypoplastic LA. Very thick deviated atrial septum with small superior patent atrial communication and multiple additional tissue folds in LA. |
| Patient 5 | Hypoplastic LA. Severely deviated septum with small superior patent atrial communication and additional tissue in LA forming muscular ridge to the LA free wall. |
| Patient 6 | Hypoplastic LA. Thick deviated septum with multiple fenestrations and a muscular ridge to the left atrial free wall. |
| Patient 7 | Hypoplastic LA. Severely deviated septum with a small inferior patent atrial communication and additional tissue in LA forming a muscular ridge to the free wall. |
| Patient 8 | Hypoplastic LA. Deviated septum with small central patent atrial communication and additional tissue fold in LA. |
| Patient 9 | Very hypoplastic and superior displaced LA that has a “Figure 8” shape with right and left pulmonary veins entering into each part and divided by a muscular ridge. Intact atrial septum. |
| Patient 10 | Extremely hypoplastic and superior displaced LA. Intact atrial septum. |

Abbreviations: LA; left atrium.

**Supplemental Table S2**: Fetal and neonatal characteristics stratified by atrial morphology pattern type.

| **Atrial septal morphology pattern** | **1** | **2** | **3** | **4** |
| --- | --- | --- | --- | --- |
|  | *n=2* | *n=9* | *n=33* | *n=10* |
| Intact atrial septum | 2 (100%) | 0 (0%) | 8 (24%) | 2 (20%) |
| Forward-to-reverse VTI ratio < 3:1 | 2 (100%) | 7 (78%) | 22 (67%) | 6 (60%) |
| Accessory channel from LA | 0 (0%) | 0 (0%) | 6 (18%) | 4 (40%) |
| Fetal atrial septal intervention performed | 1 (50%) | 3 (33%) | 5 (15%) | 3 (30%) |
| Fetal demise | 1 (50%) | 1 (11%) | 5 (15%) | 0 (0%) |
| *Fetal MRI performed* | *n=1* | *n=5* | *n=12* | *n=5* |
| Pulmonary lymphangiectasia | 1 (100%) | 2 (40%) | 4 (33%) | 2 (40%) |
| *Live born with intention to treat* | *n=0* | *n=3* | *n=9* | *n=5* |
| Atrial septal intervention prior to stage 1 palliation | - | 2 (67%) | 0 (0%) | 2 (40%) |
| 30-days mortality | - | 0 (0%) | 0 (0%) | 1 (20%) |
| 6-month mortality | - | 2 (67%) | 1 (11%) | 3 (60%) |

Abbreviations: LA; left atrium, MRI; magnetic resonance imaging, VTI; velocity time integral

**Supplemental Table S3**: Side-differences in forward-to-reverse pulmonary venous flow VTI ratios. a) Number of patients with different combinations of left-sided and right-sided measurement in 29 fetuses with bilateral measurements available on the first fetal echocardiogram in gestation. b) Number of patients with different combinations of left-sided and right-sided measurement in 9 fetuses with bilateral measurements available on the last study in gestation or before fetal atrial septal intervention.

a)

|  | **< 5:1** | **3-5:1** | **≤ 3:1** |
| --- | --- | --- | --- |
| **< 5:1** | 0 | - | - |
| **3-5:1** | 3 | 7 | - |
| **≤ 3:1** | 1 | 6 | 12 |

b)

|  | **< 5:1** | **3-5:1** | **≤ 3:1** |
| --- | --- | --- | --- |
| **< 5:1** | 0 | - | - |
| **3-5:1** | 1 | 1 | - |
| **≤ 3:1** | 0 | 3 | 4 |
